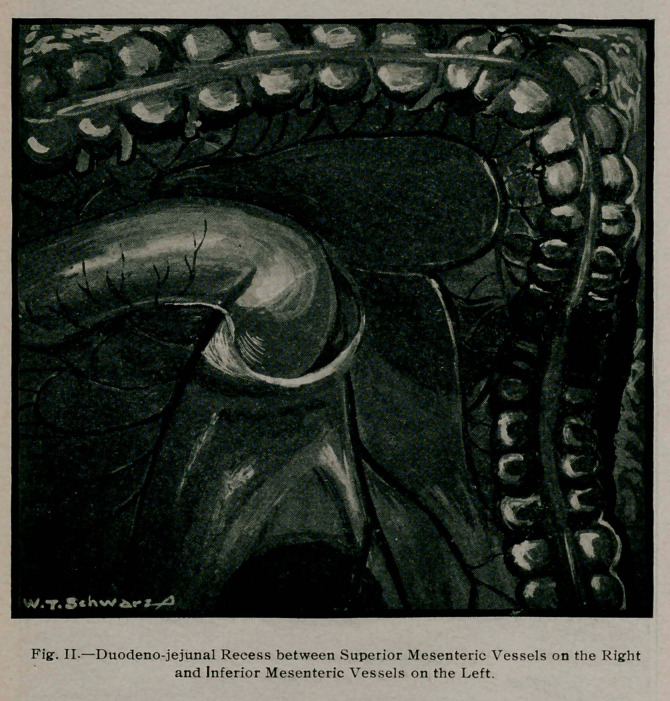# Intraperitoneal Hernia. A Case

**Published:** 1911-01

**Authors:** William L. Wallace, James S. Allen

**Affiliations:** Syracuse, N. Y.; Syracuse, N. Y.


### Intraperitoneal Hernia. A Case.

By WILLIAM L. WALLACE, M.D. and JAMES S. ALLEN, M.D.
Syracuse, N. Y.

Mr. S., aged 35, a chauffeur, came to the office March 10, 1910, complaining of recurrent attacks of severe pain in the right side of his abdomen. Family and personal history negative. He had always been well up to four or five years ago, since which time he had had severe attacks of cramp-like pains which had come suddenly, at times when running his car, and had been severe enough to force him to leave the machine and lie doubled up at the roadside. Each attack of pain had subsided very gradually. For the last year he had vomited his breakfast nearly every morning; but he had not vomited during the attacks except once or twice. He had been very constipated, requiring large amounts of cathartic medicines, but there had been no blood or mucus in the stools.

Examination showed a well nourished man with abdomen slightly rigid and considerable tenderness on the right side just above McBurney's point. Rectal examination was negative. Exploration of gall bladder and appendix was advised.

The patient was operated at Good Shepherd Hospital, Syracuse, N. Y., March 17, 1910. A small incision was made through the outer border of the right rectus. The gall bladder and ducts were normal. The appendix was slightly inflamed and was removed. Then, thinking that enough had not been found to account for the severe pains, it was decided to make further search. Meckel's diverticular trouble was thought of, and investigation of the ileum from the ileocecal valve was begun. About three inches from the valve the intestine seemed to run into a large mass and be lost. The incision was enlarged. Then presented in the wound what appeared to be another layer of peritoneum with coils of intestine showing behind. At once retroperitoneal hernia was thought of, and examination showed that all the small intestines except the first and last three inches, with the entire mesentery, were contained in a hernial sac.

The intestines passed behind a band in the free border of a fold which formed an inferior duodenal fossa, and then pushed anteriorly, distending the double layer of the inferior duodenal fold to form a sac which filled the peritoneal cavity. The sac hung loosely, connected with the posterior abdominal wall by a comparatively narrow neck or pedicle, having a mouth above and to the left which was a little larger than a silver dollar. The intestines were carefully withdrawn and when the sac was half emptied, the mass was delivered outside the abdomen. Fig. 1. The remainder of the intestines now having been withdrawn, the collapsed sac, which was then about six inches long, hung from the posterior abdominal wall just below the duodeno-jejunal flexure. The band in the neck of the sac contained no bloodvessels of any size, and the sac which was formed by a double layer of peritoneum was tied off and removed. The wound was closed without drainage. Three weeks later he had recovered and left the hospital.

Fig. 1—Hernial sac, containing intestines, withdrawn from abdomen

Internal abdominal herniæ are usually retroperitoneal. Beginning in a preëxisting fossa, a knuckle of gut dissects up the posterior parietal peritoneum with the mesenteric bloodvessels. Such a condition is found most frequently in the duodeno-jejunal region. The duodenum, it will be remembered, comes forward as the jejunum from beneath the transverse mesocolon, after passing under the superior mesenteric artery. If we pull the jejunum to the right as in Fig. 2, the small gut is actually hooked around the superior mesenteric vessels, with the duodenum behind and the jejunum in front of the duodeno-jejunal angle at the left. Thus the duodeno-jejunal region is in a natural fossa

Fig. II.—Duodeno-jejunal Recess between Superior Mesenteric Vessels on the Right and Inferior Mesenteric Vessels on the Left.

with the superior mesenteric vessels at the right and the inferior at the left. The number of minor fossæ in this region may be indefinitely multiplied, according to the folds and adhesions, Moynihan describing nine. Three forms of hernia are usual. A right duodenal hernia is formed when the gut pushes a sac to the right under the arch of the superior mesenteric vessels. A left duodenal hernia is formed when the gut pushes a sac of peritoneum to the left under the inferior mesenteric vessels. If the gut pushes a sac under the left colic artery into the transverse mesocolon, a so-called mesenteric hernia is formed.

The inferior duodenal fossa looks upward and toward the left with the blind end downward and toward the right. The margin of the double fold of peritoneum forming the fossa is usually bounded by a comparatively thick fibrous non-vascular band. A hernia into this fossa if it dissected to the left or right would be a left or right retroperitoneal hernia; if it distended the inferior duodenal fold into a sac coming out into the free peritoneal cavity between the bloodvessels, it would not be retroperitoneal, but intraperitoneal, as was this case.

Inferior duodenal fossæ are very common. In ten autopsies performed during the last few weeks the inferior duodenal fossa was found nine times.

1000 E. Genesee Street.

## Figures and Tables

**Fig. I. f1:**
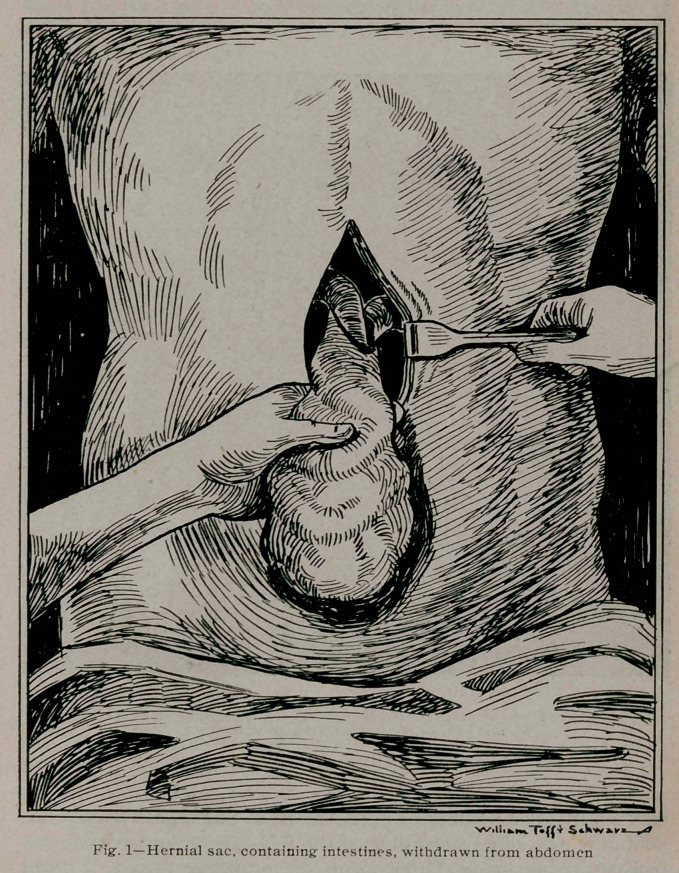


**Fig. II. f2:**